# Supplementary material for: Thioredoxin-1 protects against androgen receptor-induced redox vulnerability in castration-resistant prostate cancer
Source: Nat Commun. 2017 Oct 31;8:1204. doi: 10.1038/s41467-017-01269-x (PMC5663934; doi:10.1038/s41467-017-01269-x)
Supplement: Supplementary file 1 — Supplementary Information [file 41467_2017_1269_MOESM1_ESM.pdf]

a

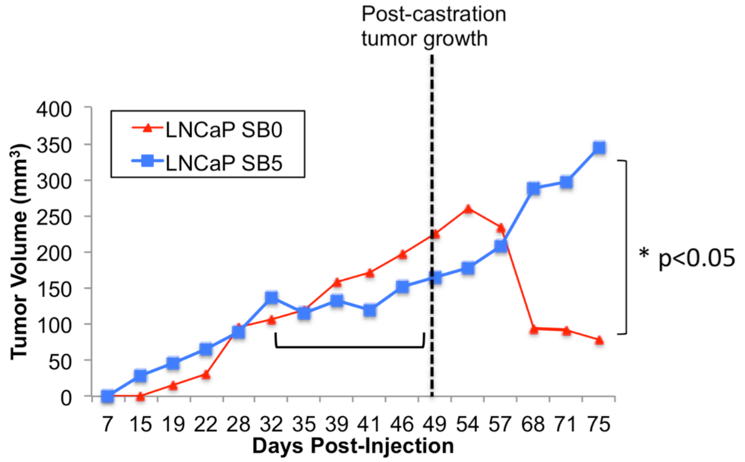

b

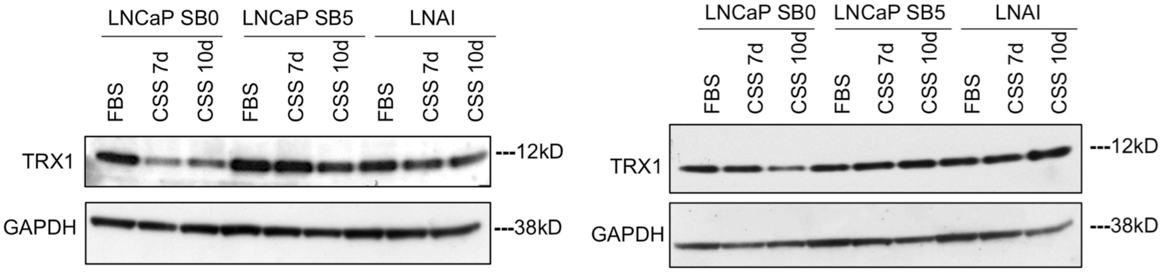

Supplementary Figure 1. LNCaP SB5 cells exhibit castration-resistant tumor growth, and sustain higher TRX1 levels under AD unlike their parental LNCaP SB0 counterparts

- **a.** Approximately $2x10^6$ cells were suspended in a 1:1 RPMI-1640 complete media:matrigel mixture and injected subcutaneously into the flanks of five-week old Nu/Nu male animals (n=11/cell line). Tumor growth was measured as described in the Methods section. Castrations were carried out on a rolling basis (indicated by the flat bracket) once tumors reached approx. 100 mm^3. The p-value was calculated on endpoint mean values per cell line using an unpaired Student's t-test.
- **b.** Examples of western blots showing TRX1 levels under FBS and indicated CSS culture conditions in LNCaP SB0, LNCaP SB5 and LNAI cells. Blots were run on independently established samples and included in the quantitation shown in Fig. 1f.

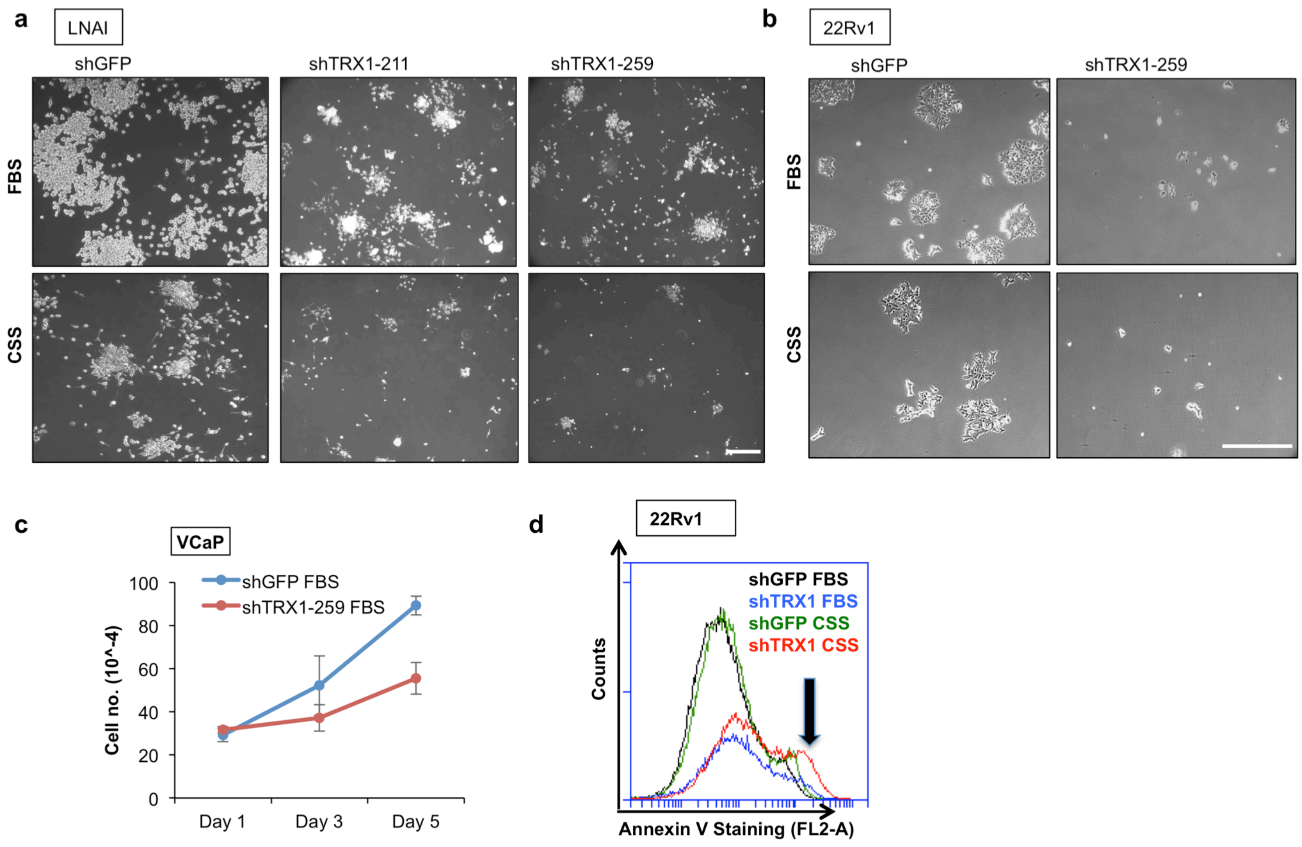

Supplementary Figure 2. TRX1 knockdown suppresses cell growth to a greater extent in CRPC cells compared to androgen-dependent PCa cells

a-b. Representative light microscopy images are shown from cells used to establish growth curves in Fig. 2 from (**a**) LNAI and (**b**) 22Rv1 transduced with either shGFP or shTRX1 constructs. Cells were cultured under the indicated conditions for 7 days. Images were acquired on a Zeiss Axiovert 40CFL microscope with an attached Axiocam ICc 5 camera. LNAI images were taken at 5X objective magnification and 22RV1 images were taken at 10X objective magnification in order to show representative effects of TRX1 knockdown with optimum clarity in the respective cell lines. Size bars, in white, indicate a 100 µm scale.

c. VCaP cells continue to divide under TRX1 knockdown. $3x10^5$ cells were plated following shRNA transduction and puromycin selection. Samples were counted on indicated days following initial plating. Data are representative of two experimental repeats, each sample run in triplicate per experiment. Error bars represent ±SD. Note VCaP cells behave similarly to LNCaP SB0 but not the CRPC lines under TRX1 knockdown (Fig. 2).

d. Annexin V staining to detect apoptotic cells. Staining was carried out in 22Rv1 cells following 48 hours of culture under denoted conditions. A rightward shift and increased peak height (indicated by the arrow) shows elevated staining. The flow cytometric profile is representative of two independent experiments.

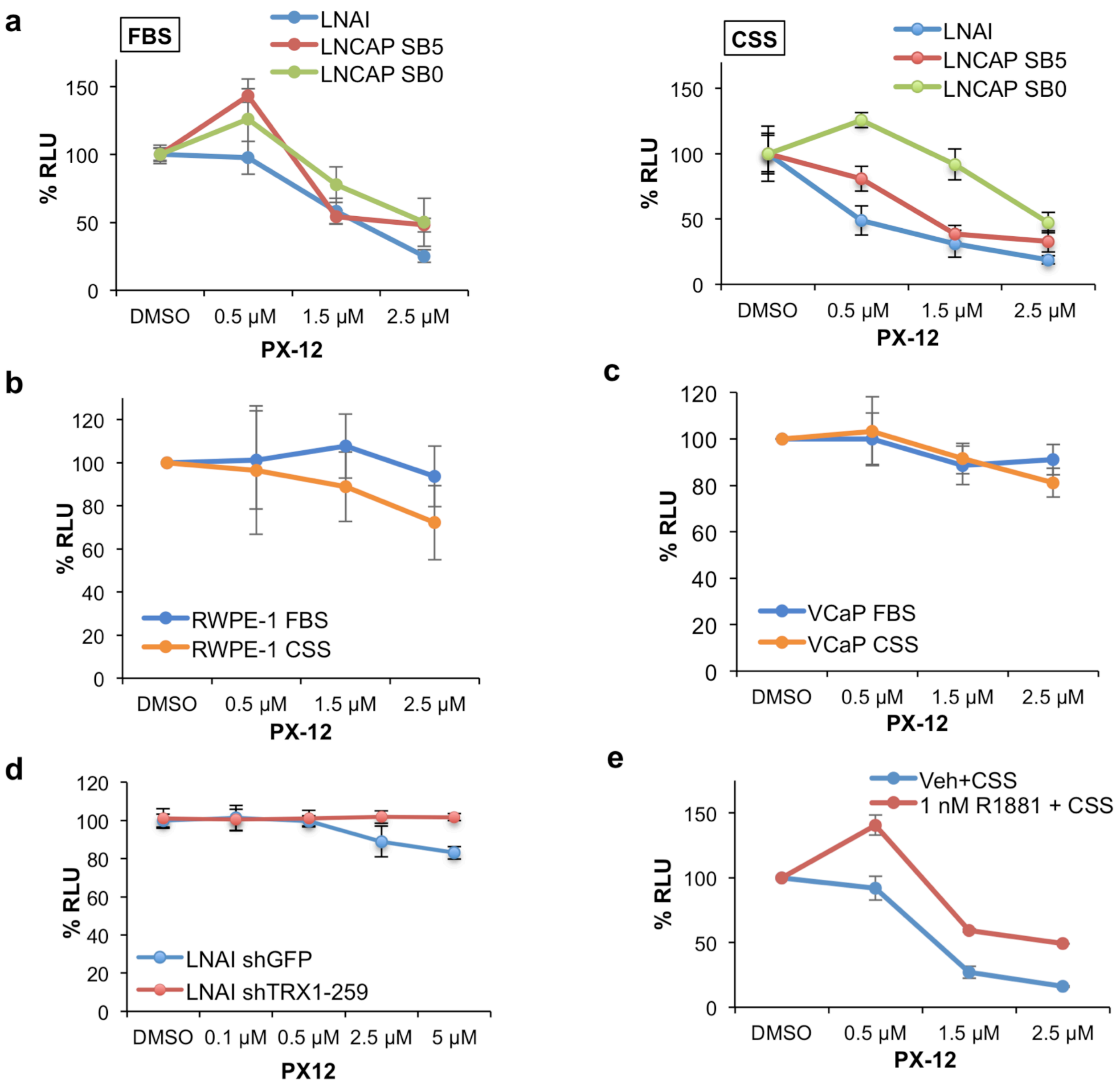

Supplementary Figure 3. PX-12 is a TRX1-specific inhibitor and sensitizes CRPC cells to AD-induced loss of viability

- **a.** For each of the indicated cell lines, cells were plated in triplicate and treated for 72 hours with DMSO or the PX-12 doses noted on the abscissa in either FBS or CSS-containing media prior to assessing viability.
- **b.** RWPE-1 cells were plated in triplicate, treated, and processed as in (a).
- **c.** VCaP cells were plated in triplicate, treated, and processed as in (a).
- **d.** LNAI cells, transduced with either shGFP or shTRX1, were plated in triplicate and treated with either DMSO or the indicated PX-12 doses for 24 hours under FBS culture conditions.
- **e.** LNAI cells were plated in triplicate and treated with either DMSO or PX12 at the indicated doses for 72 hours in CSS media, containing either vehicle (ethanol) or 1 nM R1881.

Note that data are representative of two independent experimental repeats and that the error bars represent ±SD for all panels.

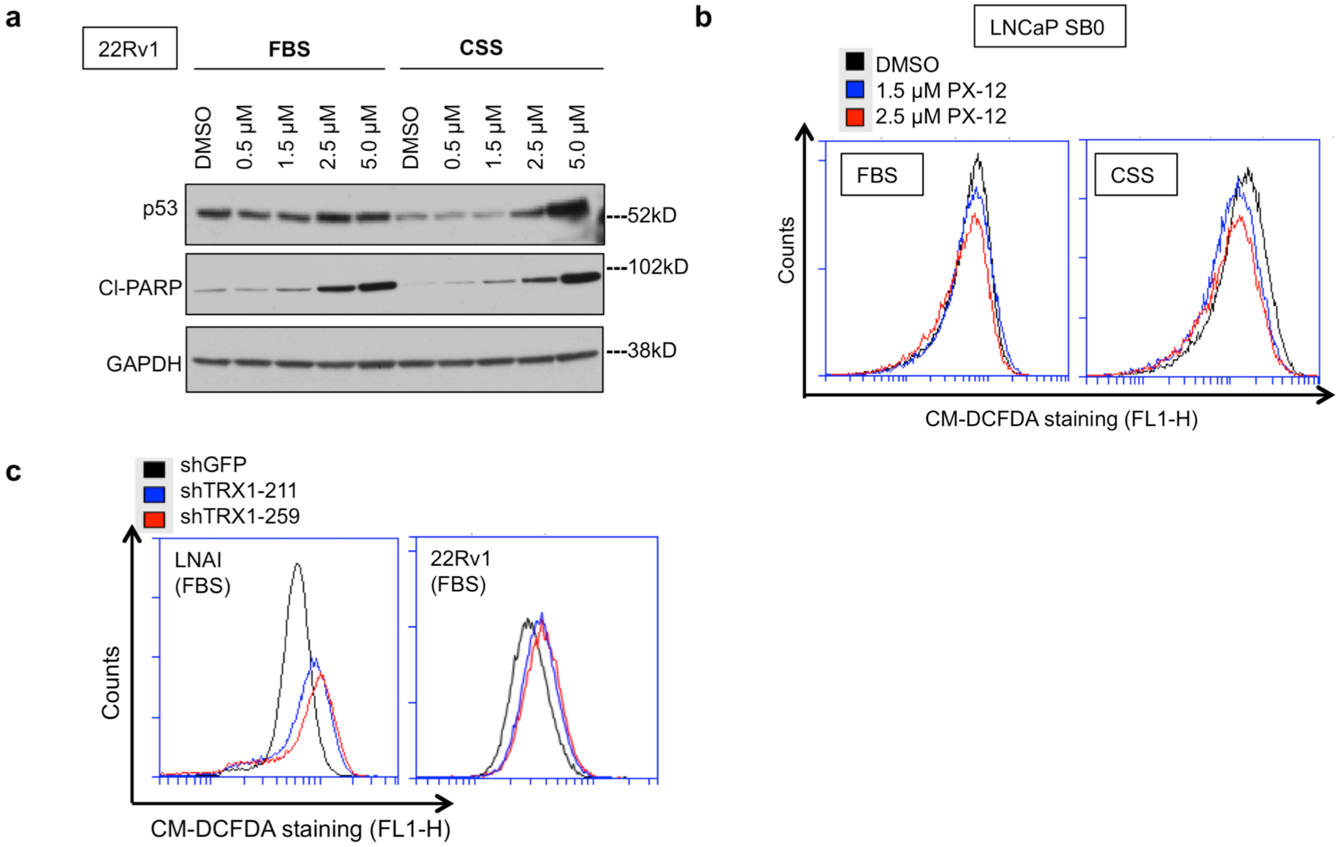

Supplementary Figure 4. TRX1 inhibition induces p53 and cleaved PARP in CRPC cells under AD, and elevates ROS in CRPC but not AD-responsive cells

- **a.** Western blot following 48 hour DMSO or PX-12 treatment of 22Rv1 cells at the indicated doses under FBS or CSS culture.
- **b.** ROS levels in PX-12-treated LNCaP SB0 cells under FBS or CSS culture. Cells were treated with PX-12 for approximately 7 hours prior to CM-H2DCF-DA staining. Note that CSS culture does not significantly alter ROS levels in LNCaP SB0 cells under PX-12 treatment. The flow cytometric profiles are representative of two independent experiments.
- **c.** Steady-state ROS levels in control shRNA and TRX1 knockdown cells. ROS levels were measured after approximately 7 days of culture in FBS media following shRNA transduction. The flow cytometric profiles are representative of two independent experiments.

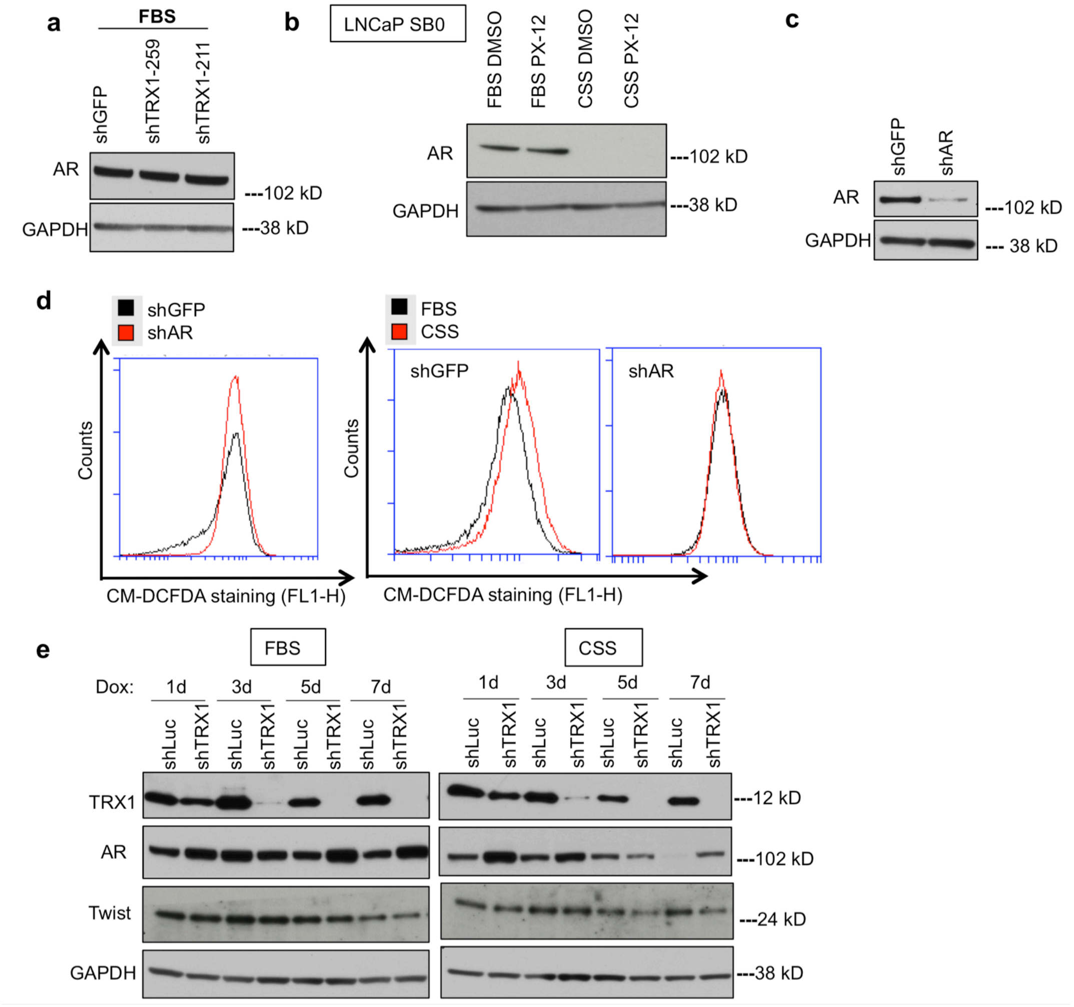

Supplementary Figure 5. **AR enhances TRX1 inhibition-induced redox stress in androgen-deprived CRPC cells**

- **a.** Western blot run on the indicated LNAI transduced cell lines under FBS culture. This is the FBS counterpart to the CSS data in Fig. 4a and run on the same blot. Note that TRX1 knockdown does not substantially alter AR expression under FBS conditions.
- **b.** Western blot for PX-12 treatment effect on AR expression in LNCaP SB0. Cells were treated with 1 μM PX-12 or DMSO for 72 hours under FBS or CSS conditions prior to being harvested for western blotting.
- **c.** Western blot showing the extent of AR knockdown in the LNAI cells used in Fig. 4.
- **d.** ROS levels in shGFP or shAR-transduced LNAI cells. Baseline levels under FBS culture are shown on the left. Relative changes within LNAI shGFP or shAR upon approximately 6 hours of CSS culture are shown on the right. The flow cytometric profiles are representative of two independent experiments.
- **e.** Western blot showing altered molecular markers in the doxycycline-inducible plko.shTRX1 system. Doxycycline (100 μg/ml) was administered to the control shLuc and shTRX1-transduced LNAI cells for the indicated number of days. These same lysates were re-run on the blot shown in Fig. 4g.

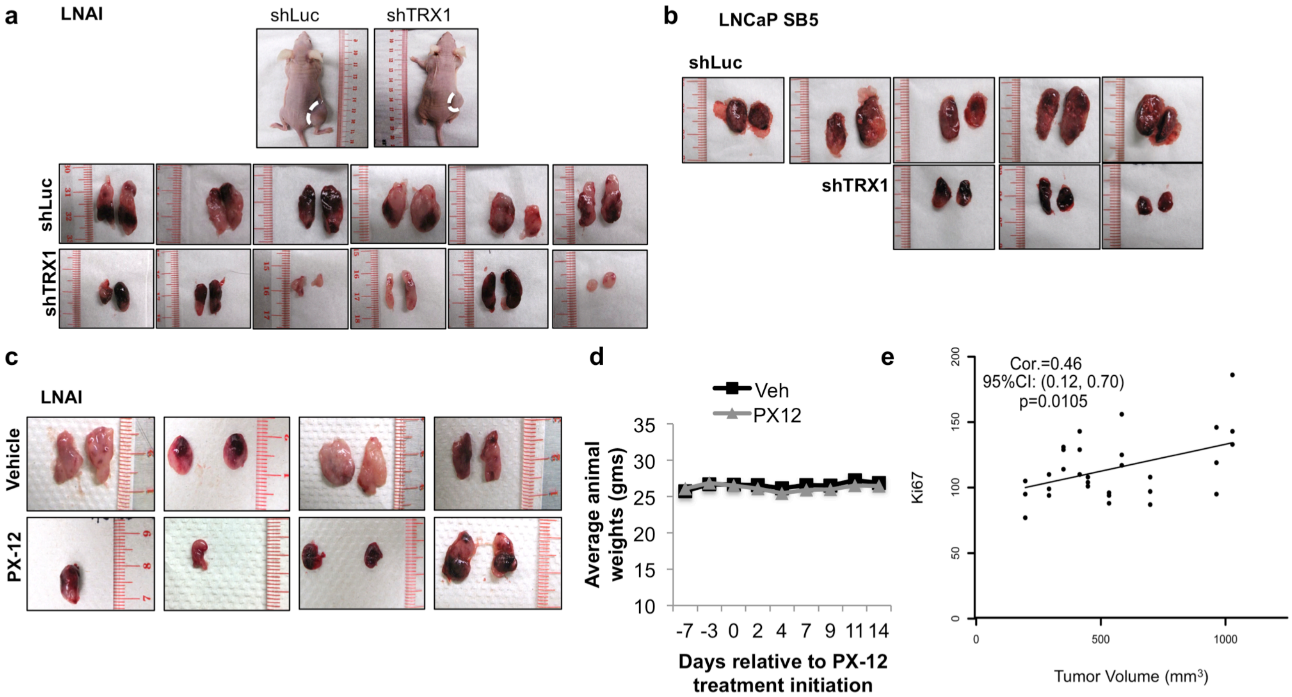

Supplementary Figure 6. **TRX1 inhibition impairs CRPC tumor formation**

- **a.** Representative images of subcutaneous LNAI shGFP and shTRX1 tumors formed in castrated male Nu/Nu mice as well as representative images of excised tumors formed by the shGFP or shTRX1 groups.
- **b.** Representative images of excised subcutaneous tumors in castrated male Nu/Nu mice from LNCaP SB5 shGFP or shTRX1 groups.
- **c.** Representative images of excised subcutaneous vehicle- or PX-12-treated LNAI tumors formed in castrated male Nu/Nu mice.
- **d.** Mean total body weights of animals in the vehicle or PX-12 treatment groups.
- **e.** Pearson's correlation analysis of Ki67 scores (number of positively-stained cells in tumor sections) plotted against sizes of the corresponding LNAI tumors from vehicle or PX-12 treated animals. Five tumors per group were analyzed, with three representative fields from each tumor being scored for Ki67 staining in a blinded-fashion. Relevant statistics are included within the figure.

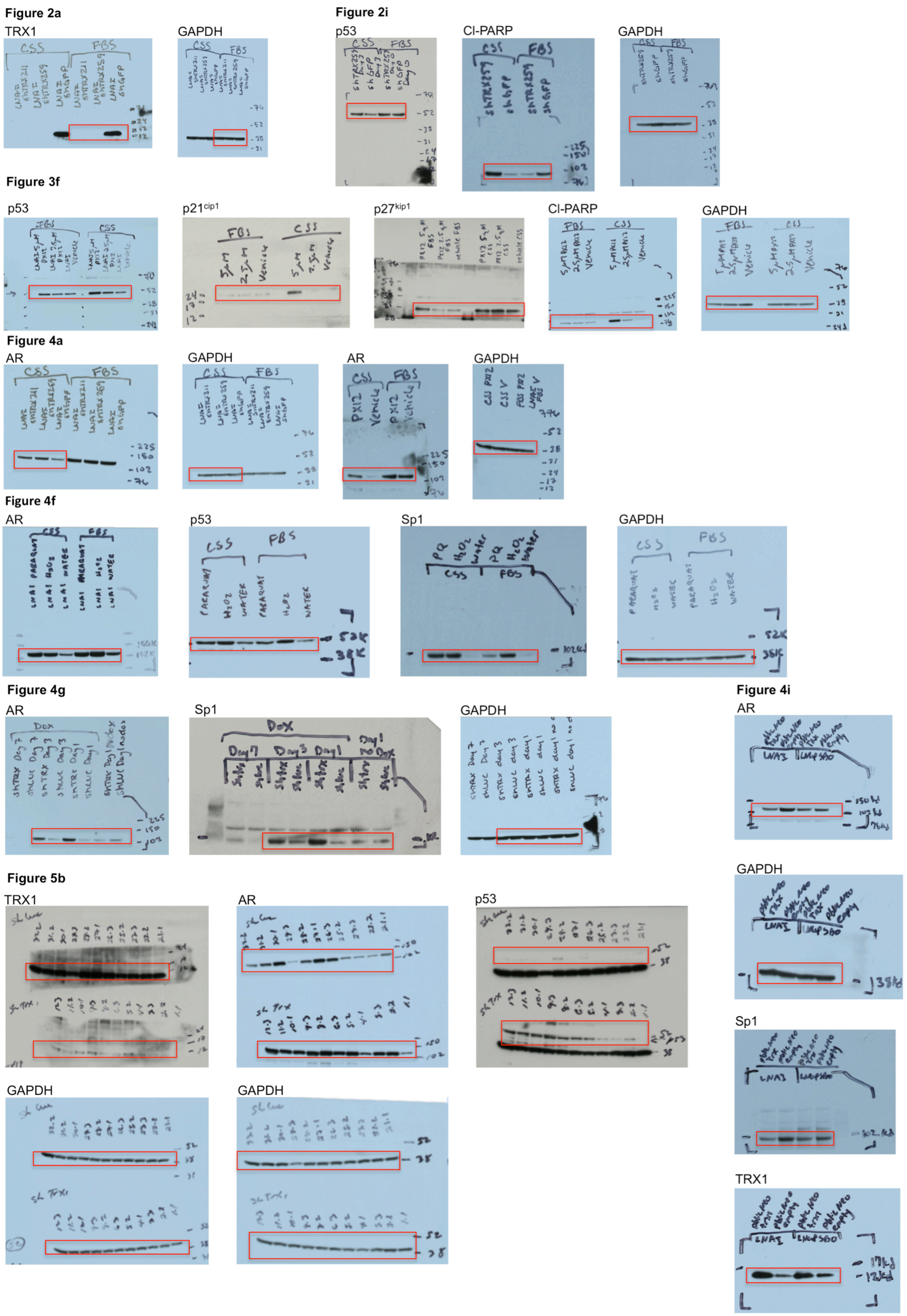

Supplementary Figure 7. Original blot images from the main and supplementary figures
Uncropped labeled original films corresponding to the western blots in the indicated figures from the main text and supplementary data are shown. The red boxes indicate cropped regions presented in the actual figures. Please note that corresponding blot images in the actual figures are horizontally inverted relative to these original films.

Figure 6f

p53

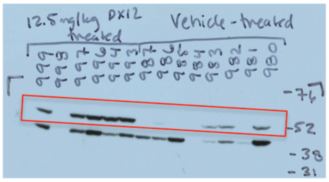

GAPDH

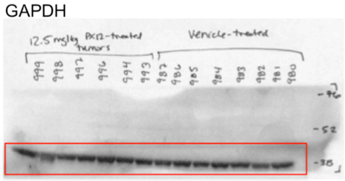

AR

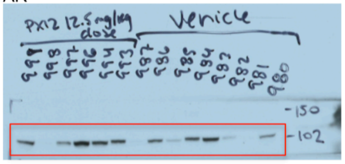

GAPDH

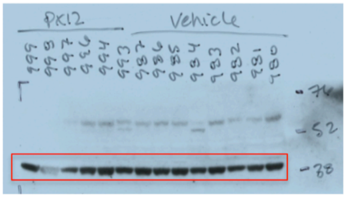

Supplementary Figure 1b

TRX1

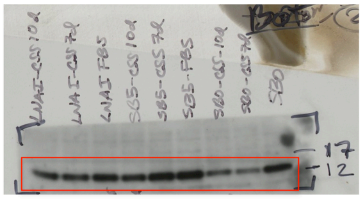

TRX1

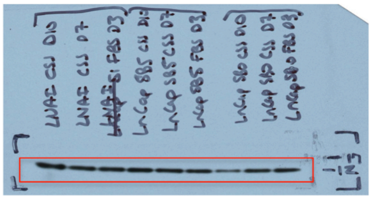

GAPDH

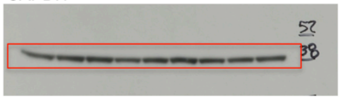

GAPDH

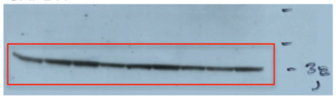

Supplementary Figure 4a

p53

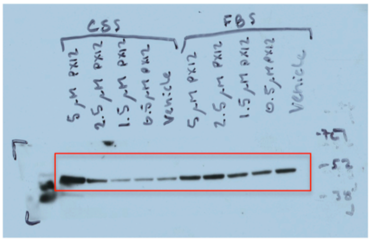

Cl-PARP

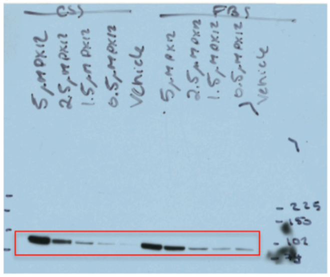

GAPDH

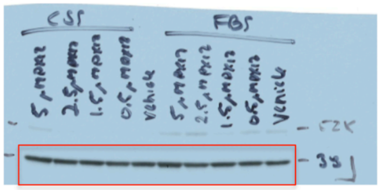

Supplementary Figure 5a

AR

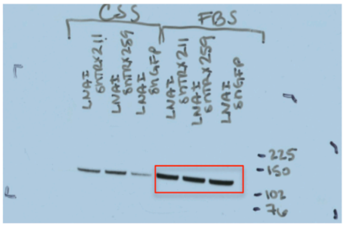

GAPDH

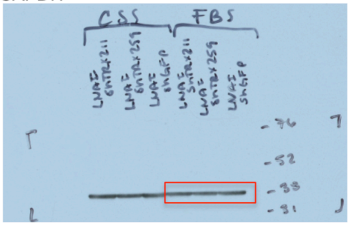

Supplementary Figure 5b

AR

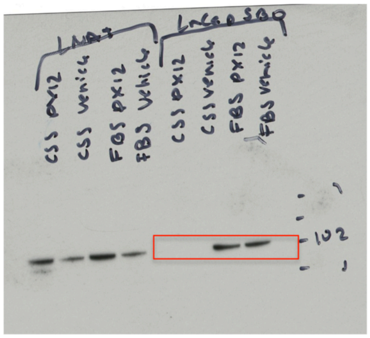

GAPDH

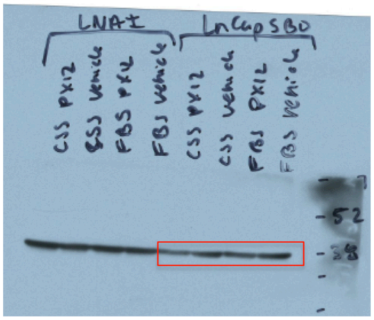

Supplementary Figure 5c

AR

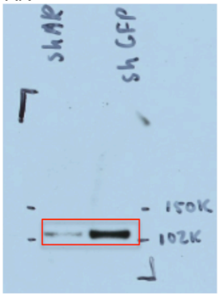

GAPDH

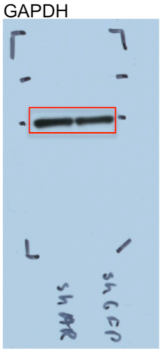

Supplementary Figure 7 (continued). Original blot images from the main and supplementary figures

Uncropped labeled original films corresponding to the western blots in the indicated figures from the main text and supplementary data are shown. The red boxes indicate cropped regions presented in the actual figures. Please note that corresponding blot images in the actual figures are horizontally inverted relative to these original films.

Supplementary Figure 5e

AR

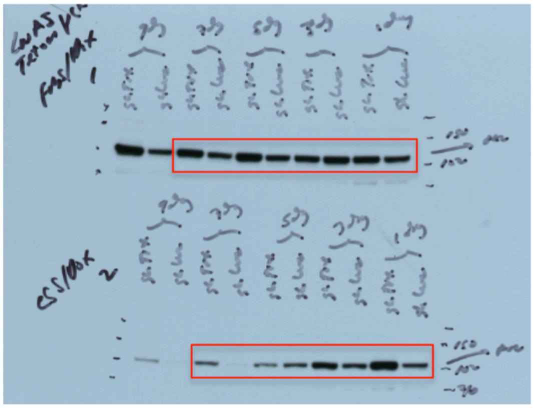

Twist

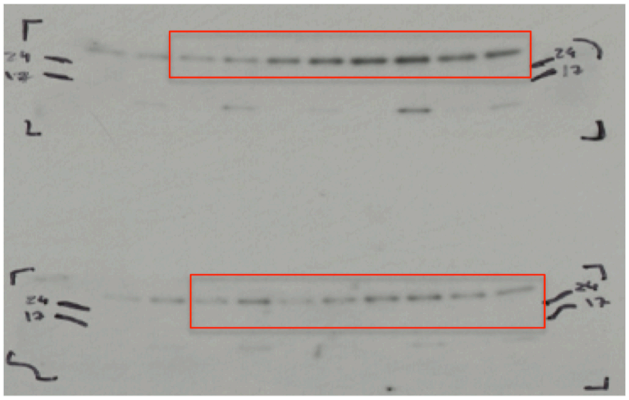

TRX1

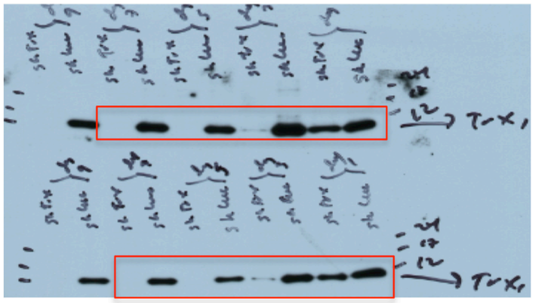

GAPDH

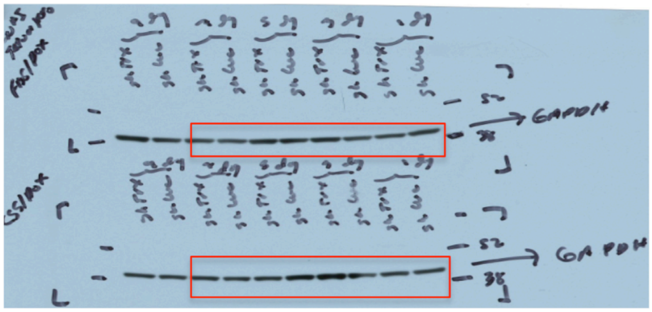

Supplementary Figure 7 (continued). Original blot images from the main and supplementary figures

Uncropped labeled original films corresponding to the western blots in the indicated figures from the main text and supplementary data are shown. The red boxes indicate cropped regions presented in the actual figures. Please note that corresponding blot images in the actual figures are horizontally inverted relative to these original films.
